# Which anthropometric equation to predict body fat percentage is more strongly associated with maximum oxygen uptake in adolescents? A cross-sectional study

**DOI:** 10.1590/1516-3180.2022.0437.R1.07022023

**Published:** 2023-05-12

**Authors:** Eliane Cristina de Andrade Gonçalves, Nelson Nardo, Michele Caroline de Souza Ribas, Diego Augusto Santos Silva

**Affiliations:** I PhD. Assistant Professor, Department of Physical Education, Universidade Estadual de Maringá (UEM), Maringá (PR), Brazil.; II PhD. Associate Professor, Department of Physical Education, Universidade Estadual de Maringá (UEM), Maringá (PR), Brazil.; III PhD. Assistant Professor, Department of Physical Education, Universidade Federal de Santa Catarina (UFCA), Florianópolis (SC), Brazil.; IV PhD. Associate Professor, Department of Physical Education, Universidade Federal de Santa Catarina (UFCA), Florianópolis (SC), Brazil. Associate Researcher, Faculty of Health Sciences, Universidad Autónoma de Chile, Providencia, Chile.

**Keywords:** Fat body, Exercise, Physical fitness, Association, Lifestyle, Physical activity, Adolescent health, Overweight

## Abstract

**BACKGROUND:**

Identifying the relationship between maximum consumption of oxygen and body fat percentage is important due to increased cardiovascular risk factors.

**OBJECTIVE:**

This study aimed to verify the association between body fat percentage determined by three predictive equations using anthropometric measures (Lohman, Boileau, and Slaughter) and maximum oxygen uptake (VO_2_max). We also aimed to estimate the capacity of these equations for explaining VO_2_max variations in adolescents according to sex.

**DESIGN AND SETTING:**

This was a cross-sectional study conducted in high schools in São José, Southern Brazil.

**METHODS:**

This study included 879 adolescents (14–19 years) from Southern Brazil. Aerobic fitness was assessed using the modified Canadian Aerobic Fitness Test. The independent variable was body fat percentage predicted by the Lohman, Boileau, and Slaughter equations. Analyses adjusted for sociodemographic variables, physical activity level, and sexual maturation were performed with P value < 0.05.

**RESULTS:**

All anthropometric prediction equations used to estimate body fat percentage explained VO_2_max variations in adolescents. In male adolescents, both regression models based on the Boileau et al.^12^ and Lohman^10^ equations revealed higher explanatory power for VO_2_max (20%) compared with that based on the Slaughter et al.^13^ equation (19%). In female adolescents, the model based on the anthropometric equation of Slaughter et al.^13^ showed the greatest explanatory power for VO_2_max (18%).

**CONCLUSION:**

The inverse relationship between VO_2_max and body fat intensifies the need for effective intervention programs that prioritize maintenance of appropriate body fat and aerobic fitness levels because inadequate levels of both factors result in negative health consequences.

## INTRODUCTION

Aerobic fitness is defined as the ability to supply oxygen to the muscles for generating energy during physical exercise.^1^ Although it is a physiological determinant of running performance, aerobic fitness is not only restricted to sports performance, but also used as a diagnostic measure of health and physical exercise prescription.^1^

An adequate aerobic fitness level is an important marker of health in children and adolescents and strongly associated with disease prevention including obesity, hypertension, and diabetes at all stages of life.^2,3^ On the other hand, low aerobic fitness levels have been reported to be associated with increased cardiovascular risk factors that may occur during adolescence, such as hypertension, insulin resistance, and metabolic risk factors.^2^

Research conducted in 2015 throughout Brazil indicated that approximately three million adolescents aged 13–17 years were overweight.^4^ The world estimates reported that in the year 2025, approximately 75 million children and adolescents will be overweight and obese.^5^ In Latin America, approximately 21 million adolescents (between 2008 and 2013) and one-third of adolescents in the United States (in 2003 and 2004) had body fat above healthy recommended levels. This is of concern because overweight was found to be a risk factor for cardiovascular and pulmonary diseases; diabetes mellitus; biliary disorders; and certain types of cancer.^6,7^

The gradual decrease in aerobic fitness levels in adolescents^8^ is even more worrying when associated with excess body adiposity. Studies have identified that one of the possible explanations for various cardiovascular changes and occurrence of chronic diseases in individuals with overweight may be related to low aerobic fitness levels.^2,3^ These findings seem independent of the method used to predict excess adiposity,^9^ regardless of the technique used to measure body composition, excess body fatness is a predictor of low aerobic fitness levels.

One way to identify excess body adiposity is through anthropometric measurement.^10^ This practice is widely used in epidemiological studies because it is easy to apply and has low cost and good validity indexes compared with more accurate methods.^3^ Since body fat percentage prediction equations using skinfolds are non-invasive methods that systematically measure body dimensions and proportions, they are well accepted and widely used in population research. These equations help detect changes in body pattern; health conditions; and performance and functional capacity.^11^

Analyzing the explanatory capacity of body fat percentage prediction equations for estimating maximum oxygen uptake (VO_2_max), it is possible to identify whether equations that use only two skinfolds (triceps and subscapular) could be sufficient to predict VO_2_max variation in adolescents. In addition, this study could allow for the identification and comparison of the relationship of each equation with aerobic fitness, thus helping to choose the most efficient equation according to sex.

## OBJECTIVE

This study aimed to verify the association between body fat percentage analyzed by three different anthropometric predictive equations^10,12,13^ and VO_2_max and estimate the explanatory capacity of these equations to explain VO_2_max variations in adolescents according to sex.

## METHODS

### Participants

This cross-sectional, school-based research was part of the macro-project entitled “Brazilian Guide for the Assessment of Physical Fitness Related to Health and Life Habits - Stage I”. The study was approved by the Human Research Ethics Committee of the Universidade Federal de Santa Catarina on 14^th^ August 2014 (CAAE Protocol: 33210414.3.0000.0121) and conducted between August and November 2014.

The study population recruited 5,182 high school students aged 14–19 enrolled in high schools in the city of São José southern Brazil, distributed across 11 eligible schools and 170 high school classes.

The sample process was determined at two stages: (i) stratified by state public high schools (n = 11) and (ii) class conglomerates considering study shift and school grade (n = 170 classes). At stage 2, all students enrolled in high schools who were present in the classroom on days of data collection were invited to participate in the study.

Regarding sample size calculation, unknown prevalence for the outcome (50%), tolerable error of five percentage points, 95% confidence level, and design effect of 1.5 were considered, adding 20% for losses and refusals and a further 20% for the association study. A sample size of 751 adolescents was found to be large enough in this study. However, due to conglomerate sampling, all students were invited to participate in the survey (1,132 students).

Eligibility was defined as being: enrolled in the state education network, present in classroom on the day of data collection, and aged 14–19 years. All adolescents who participated in the survey provided written informed consent signed by parents or responsible persons (for the participants under 18 years of age) or the participants themselves (if aged above 18 years). Students who did not want to participate in the research were considered refusals. Moreover, those with incomplete questionnaires or who did not undergo one or more physical tests were considered losses.

### Measures

The dependent variable VO_2_max was estimated using the modified Canadian Aerobic Fitness Test (mCAFT),^14^ validated in comparison with indirect calorimetry in men and women aged 15–69 years.^15^ Considering the test, adolescents had to complete one or more stages of three minutes each, where they had to go up and down two steps (20.3 centimeters). The stage and initial velocity were predetermined according to the participants’ sex and age. The rhythm for performing the steps within each stage of the test was performed with musical cadence, indicating that when the adolescent should go up and down the steps.^14^ The test was finalized only when the participants reached 85% of the maximal heart rate (determined by the formula 220 - age),^14^ which was measured using a Polar H7 Bluetooth heart rate monitor (Kempele, Finland). If the participants did not reach 85% of the maximal heart rate at a given stage, a new stage was started shortly after the last stage until 85% of the maximum heart rate was reached at the end of the test. The final stage of the test was considered the one in which the adolescent was able to perform completely: if 85% of the maximum heart rate was reached during a certain stage, the stage prior to the adolescent ’s performance was recorded as the final stage.

The oxygen expenditure during exercise and reference values for determination of the beneficial health zone for aerobic fitness were determined by the Canadian battery.^14^ The aerobic fitness score equation determined by the Canadian battery was: *Score = 10 [17.2 + (1.29 x Oxygen expenditure) - (0.09 x weight in kg) - (0.18 x age in years)*.

The score was divided by 10 to obtain the value estimated for VO_2_max,^14^ which was continuously analyzed.

The independent variables were the anthropometric equations used for predicting body fat percentage. The Boileau et al.,^12^ Slaughter et al.^13^ and Lohman^10^ equations (Table 1) were used. The Pires-Neto and Petroski^16^ (the information is at https://osf.io/4evdf/) constants were used for the Lohman^10^ equation. Socio-demographic data were collected using self-administered questionnaires. Skin color was auto-referred according to the Brazilian Institute of Geography and Statistics^17^ and dichotomized in “White” and “Brown/Black/Yellow/Indigenous”. Age was categorized as “14–16 years” and “17–19 years.” Economic level was identified using the questionnaire of the Brazilian Association of Research Companies^18^ and dichotomized as “High” (“A1,” “A2,” “B1,” “B2”) and “Low” (“C1”; “C2”; “D”; “E”).

**Table 1.** Equations for predicting the percentage of body fat in children and adolescents

Reference	Year	Sex	Predictive equation’s
Boileau et al.^12^	1985	Male	%FAT = 1.35 (TR + SE) – 0.012 (TR+SE)^2^ - 4,4
		Female	%FAT = 1.35 (TR + SE) – 0.012 (TR + SE)^2^ - 2,4
Lohman^10^	1986	Male and female (all ages)	%BF = 1.35 (TR + SE) − 0.012 (TR + SE)^2^ - I^a^
Slaughter et al.^13^	1988	Male ∑BF < 35mm (all ages)	%BF = 1.21 (TR + SE) − 0.008 (TR + SE)^2^ + I^b^
		Female ∑BF < 35mm (all ages)	%BF = 1.33 (TR + SE) − 0.013 (TR + SE)^2^ - 2.5
		Male ∑BF ≥ 35 mm (all ages)	%BF = 0.783 (TR+SE) + 1.6
		Female ∑BF ≥ 35 mm (all ages)	%BF = 0.546 (TR+SE) + 9.7

%FAT = fat percentage; TR = triceps skinfold; SE = subscapular skinfold; %BF = body fat percentage; I^a^ = Intercept based on sex, age and ethnicity proposed by Lohman;^10^ ∑BF = sum of body fat; I^b^ = Intercept substitutions based on maturation and ethnicity for boys proposed by Slaughter et al. (1988).

Physical activity level was assessed using the following question: “During the past seven days, on how many days were you physically active for at least 60 minutes a day?” Adolescents who practiced physical activity five days or more a week were classified as “physically active (≥ 300 minutes per week)” and less than five days/week as “little physically active (< 300 minutes per week).^19^”

Sexual maturation was evaluated according to the criteria proposed by Tanner,^20^ validated and reproducible for the Brazilian population.^21^ Stages were determined by self-assessment (figures) of breast (female participants) and genital (male participants) development after individual and previous explanations of the instrument by the researcher, always of the same sex as the adolescent. Due to the low frequency of adolescents in the pre-pubertal stage (0.2%), categories were “Pre-pubertal/Pubertal” and “Post-pubertal.” This variable was fitted in the multivariate analysis in discrete continuous form.

### Statistical analysis

The mean values, standard deviations, and frequency distributions were used for the descriptive analysis of the variables. Data distribution normality was tested using asymmetry and kurtosis analyses. The highest asymmetry value was for the body mass index (asymmetry = 1.2), and the highest kurtosis value was for the subscapular skinfold (kurtosis = 2.3). The other variables exhibited asymmetry and kurtosis values close to zero. According to the literature, such asymmetry and kurtosis values referred to normal data distribution.^22^ Thus, Student’s t-test was applied to verify differences between means according to sex. Pearson’s correlation was used to verify the relationship between the anthropometric equations to predict body fat percentage and VO_2_max according to sex.

Simple and multiple linear regression analyses were used to identify the relationship between the anthropometric equations for predicting body fat percentage and VO_2_max. Using multiple linear regression analysis, a model was constructed for each equation separately and adjusted for sociodemographic factors (skin color, age, and economic level); physical activity level; and sexual maturation. The significance level was set to 5%. Analyses were performed using the SPSS version 22.0 (IBM, New York, United States), considering the design effect and sample weight, which were stratified by sex.

## RESULTS

Of the 1,132 adolescents evaluated, 253 were excluded from the study because they did not perform the aerobic fitness test. Therefore, a total 879 adolescents with a mean age of 16.22 ± 1.14 years were finally included in the study. The mean height and VO_2_max were significantly higher in boys than in girls (Table 2). The mean triceps and subscapular skinfold, sum of the triceps and subscapular skinfold values, and body fat percentage predicted by the three anthropometric equations were significantly higher in girls than in boys (Table 2). In both sexes, VO_2_max was negatively correlated with body fat percentage, as estimated using the three equations analyzed (Figure 1).

**Table 2.** Total and stratified by sex of the mean and standard deviation of age, height, body mass, anthropometric indicators and VO_2_max of adolescents

Variables	Total sample	Males	Females	P value	Cohen’s d
	Mean ± SD	Mean ± SD	Mean ± SD		
Age (years)	16.22 ± 1.14	16.28 ± 1.19	16.16 ± 1.10	0.15	0.10
Height (cm)	166.56 ± 8.81	172.59 ± 7.35	161.17 ± 6.09	< 0.01*	1.69
Body mass (kg)	61.67 ± 12.20	65.43 ± 12.07	58.31 ± 11.32	0.25	0.60
BMI (kg/m^2^)	22.16 ± 3.72	21.89 ± 3.44	22.41 ± 3.95	0.25	0.14
TR (mm)	14.94 ± 7.34	10.75 ± 5.13	18.70 ± 6.99	< 0.01*	1.29
SE (mm)	13.32 ± 6.73	10.76 ± 4.86	15.60 ± 7.33	< 0.01*	0.77
ΣTR+SE (mm)	28.26 ± 13.49	21.51 ± 9.53	34.30 ± 13.66	< 0.01*	1.08
Boileau equation’s^12^	23.05 ± 7.42	18.02 ± 5.87	27.57 ± 5.51	< 0.01*	1.67
Lohman equation’s^10^	21.21 ± 7.53	15.99 ± 5.87	25.89 ± 5.52	< 0.01*	1.73
Slaughter equation’s^13^	22.82 ± 10.57	15.69 ± 8.32	29.23 ± 7.96	< 0.01*	1.66
VO_2_max (ml/kg/minutes)	38.80 ± 5.83	42.68 ± 5.34	35.33 ± 3.66	< 0.01*	1.60

SD = standard deviation; BMI = body mass index; TR = triceps skinfold; SE = subscapularis skinfold; ΣTR + SE: sum of triceps and subscapularis skinfolds; VO_2_max = maximum oxygen uptake; *P ≤ 0.05 (Student’s t test).

Using simple and multiple linear regression analyses, it was found that as body fat percentage increased, regardless of the anthropometric prediction equation,^10,12,13^ VO_2_max values of adolescents of both sexes decreased. The male participants presented standardized β values of -0.41, -0.41 and -0.28 for Boileau et al.,^12^ Lohman^10^ and Slaughter et al.^13^ equations, respectively. The magnitude of decrease in VO_2_max in female participants was -0.26, -0.26, and -0.19, for the Boileau et al.,^12^ Lohman^10^ and Slaughter et al.^13^ equations, respectively (Table 3).

**Table 3.** Simple and multiple linear regression for the association between VO2max and percentage of body fat analyzed by different anthropometric equation in boys and girls

Variables	Male										
	Simple		Multiple^†^			Multiple^§^			Multiple^‡^		
	β	CI 95%	β	CI95%	R^2^						
					0.20			0.20			0.19
Skin color	0.80	(-0.14; 1.76)	0.88	(-0.18; 1.95)		0.89	(-0.17; 1.96)		0.92	(-0.14; 2.00)	
Age	0.48	(-0.44; 1.41)	0.37	(-0.65; 1.40)		0.19	(-0.84; 1.22)		0.23	(-0.79; 1.27)	
Economic level	-0.01	(-1.16; 1.14)	-0.16	(-1.33; 1.00)		-0.16	(-1.33; 1.00)		-0.14	(-1.32; 1.03)	
Physical activity level	**-1.19**	**(-2.22; -0.16)**	**1.55**	**(-2.68; -0.42)**		**-1.55**	**(-2.68; -0.42)**		-1.54	(-2.67; -0.40)	
Sexual maturation	**-1.31**	**(-2.38; 0.25)**	**-1.43**	**(-2.63; -0.24)**		**-1.43**	**(-2.62; -0.23)**		-1.35	(-2.55; -0.15)	
Boileau equation’s^*12^	**-0.41**	**(-0.49; -0.33)**	**-0.38**	**(-0.47; -0.29)**							
Lohman equation’s^*10^	**-0.41**	**(-0.49; -0.33)**				**-0.38**	**(-0.47; -0.29)**				
Slaughter equation’s^*13^	**-0.28**	**(-0.34; -0.22)**							**-0.27**	**(-0.33; -0.20)**	
	Female										
Skin color	0.73	(0.09; 1.36)	0.69	-0.01; 1.40	0.14	0.69	-0.01; 1.40	0.14	0.74	0.04; 1.43	0.18
Age	-0.31	**(-0.93; 0.31)**	**-0.40**	**(-1.10; 0.29)**		**-0.53**	**(-1.23; 0.16)**		-0.87	-1.56; -0.18	
Economic level	0.36	(-0.32; 1.05)	0.45	-0.25; 1.16		0.44	-0.26; 1.15		0.55	-0.14; 1.24	
Physical activity level	-0.76	**(-1.57; 0.03)**	**-0.47**	**(-1.37; 0.42)**		**-0.46**	**(-1.36; 0.42)**		-0.44	-1.31; 0.43	
Sexual maturation	-0.18	**(-0.87; 0.50)**	**-0.44**	**(-1.20; 0.31)**		**-0.45**	**(-1.20; 0.31)**		**-0.19**	**(-0.95; 0.56)**	
Boileau equation’s^*12^	-0.26	**(-0.32; -0.21)**	**-0.24**	**(-0.30; -0.17)**							
Lohman equation’s^*10^	-0.26	**(-0.32; -0.20)**				**-0.23**	**(-1.21; 0.31)**				
Slaughter equation’s^*13^	-0.19	**(-0.23; -0.15)**							**-0.18**	**(-0.23; -0.14)**	

B = slope coefficient; CI = confidence interval 95%; R^2^ = determination coefficient; *Adjusted analysis for skin color, age, economic level, physical activity level and sexual maturation. ^†^Adjusted model for percentage of body fat estimated by Boileau equation’s;^12 §^Adjusted model for percentage of body fat estimated by Lohman equation’s;^10 ‡^Adjusted model for percentage of body fat estimated by Slaughter equation’s.^13^

Multiple linear regression analysis revealed that regardless of sociodemographic factors (skin color, age, and economic level); physical activity level; and sexual maturation, anthropometric equations used to predict body fat percentage presented explanatory power for VO_2_max above 14% (R^2^) in both sexes. In male participants, both regression models based on the Boileau et al.^12^ and Lohman^10^ equations presented explanatory power for VO_2_max of 20%, which was greater than the regression model based on the Slaughter et al.^13^ equation (19%). The regression models based on the Boileau et al.^12^ and Lohman^10^ equations presented greater explanatory power for VO_2_max. In female participants, the equation that presented the highest explanatory power (R² = 0.18) for VO_2_max was proposed by the Slaughter et al.^13^ equation (Table 3).

## DISCUSSION

All anthropometric prediction equations used to estimate body fat percentage explained the VO_2_max variations in adolescents in the present study. In male participants, both regression models based on the Boileau et al.^12^ and Lohman^10^ equations presented higher explanatory power for VO_2_max compared with the regression model based on the Slaughter et al.^13^ equation. Therefore, the models with the Boileau et al.^12^ and Lohman^10^ equations were those that obtained the greatest explanatory power (R²) for VO_2_max. In female participants, the model with the Slaughter et al.^13^ anthropometric equation obtained the greatest explanatory power for VO_2_max.

The inverse relationship between body fat and VO_2_max could be explained by the fact that individuals with higher amounts of body fat might tend to experience locomotion difficulties, which results in less frequent walking and stability during walking and/or running.^23^ These aspects influence the movement economy, resulting in greater energy expenditure and precipitated fatigue during low-intensity activities.^24^

The three anthropometric equations used to predict body fat percentage^10,14,15^ were derived from the same sample,^25^ and this may explain why the differences between them in the explanation of VO_2_max in adolescents in the present study were not greater. The original study that proposed these prediction equations involved 310 American, Caucasian, and African children, adolescents, and adults. This original study was conducted in Illinois, United States, and replicated in Arizona, United States.^10,14,15^ However, the equations differ among themselves. The first equation was proposed by Boileau et al.^12^ and was established to cover the age range between 8 and 29 years, being stratified by sex (the information is at https://osf.io/4evdf/). The Lohman^10^ equation (the information is at https://osf.io/4evdf/) included an age range between 7 and16 years and used constants different from that in the Boileau et al.^12^ equation. The anthropometric equation for the prediction of body fat percentage proposed by Slaughter et al.^13^ covered the age group of 8–18 years (the information is at https://osf.io/4evdf/). However, this equation, unlike the Boileau et al.^12^ and Lohman^10^ equations, considered the sexual maturation stage in children and adolescents in addition to skin color and sex.

The models used for body fat prediction created by Boileau et al.^12^ and Lohman^10^ were the equations that obtained greater explanatory power for VO_2_max variations in boys in the present study. In girls, the Slaughter et al.^13^ equation was that best explained VO_2_max variations. Possible justification for these findings is that the Slaughter et al.^13^ equation was the only one of the three equations that used variables of sexual maturation and the puberty process in the prediction model and. Consequently, sexual maturation seems to influence more female adolescents.^26^ The reason is that, in addition to girls being at greater risk for early pubertal development, considering that the prevalence of delayed puberty was more common in male participants, female children and adolescents tended to have higher fat mass levels and higher leptin levels during childhood.^26^ Plasma leptin concentrations (responsible for body weight and energy balance regulation) are related to changes in VO_2_max and body composition during the puberty process.^27^

In male participants, leptin concentrations decrease throughout the pubertal period. However, VO_2_max increases throughout puberty to adulthood due to the higher concentration of fat-free mass in boys, according to the oxidative potential of muscle fibers.^28^ The opposite occurs in female individuals because, during puberty, girls gain fat mass, leading to increased leptin concentrations.^26^ In addition, VO_2_max increases in girls only at the onset until the end of puberty, with no change from late puberty to adulthood, as they have higher body fat concentration compared with boys.^29^ In this sense, the Boileau et al.^12^ and Lohman^10^ equations responded better to male participants because they did not comprise the variable of sexual maturation, unlike the Slaughter et al.^13^ equation, which seems to be more suitable for female individuals when the purpose is to identify associations with aerobic fitness.

The use of equations that consider only two skinfolds (triceps and subscapula) may be a limitation of this study. However, it is important to stress that the use of only two skinfolds to identify the body fat percentage makes the application simple with lower chance of error. In addition, the fact that VO_2_max was estimated using a submaximal test may be another limitation, considering that the use of submaximal protocols to estimate VO_2_max is less precise compared with maximum protocols. However, submaximal tests are more practical to apply to samples with a larger number of individuals.^30^ In addition, submaximal indirect tests using heart rate may be used to assess VO_2_max in adolescents with low physical fitness or in those who do not support maximum-effort tests.^3^

Regardless of the equation used to predict body fat percentage in this study,^10,14,15^ all equations were able to explain VO_2_max variations in adolescents. This intensifies the need for effective intervention programs that prioritize the maintenance of satisfactory body fat and aerobic fitness levels, considering that both factors result in inadequate levels that cause negative consequences and health damage, such as a predisposition to development of cardiovascular diseases. In addition, sexes responded differently to each prediction equation when associated with VO_2_max. Therefore, actions should be taken in a cautious and sex-specific manner, considering factors that directly influence VO_2_max and body fat differently in male and female individuals, such as sexual maturation.

## CONCLUSIONS

All anthropometric prediction equations used to estimate body fat percentage explained VO_2_max variations in adolescents in the present study. In male participants, the Boileau et al.^12^ and Lohman^10^ equations showed the greatest explanatory power for VO_2_max. In female participants, the model with the Slaughter et al.^13^ anthropometric equation revealed the greatest explanatory power for VO_2_max.
